# Retraction: Breast Tumor Cells with *PI3K* Mutation or *HER2* Amplification Are Selectively Addicted to Akt Signaling

**DOI:** 10.1371/journal.pone.0341208

**Published:** 2026-01-21

**Authors:** The PLOS One Editors

Following the publication of this article [1], concerns were raised about Figs 1-3 and S1.

Specifically,

- In Fig 1A, after adjusting color levels, there appears to be horizontal discontinuity left and right from the p-Akt(T308) band in lane 8,
- In Fig 2A HCC1806 (*PIK3CA* wt) the following bands appear more similar than would be expected from independent samples:
  - p-GSK3β(S9): 0 hour and 1 hour
  - p-p70S6K(T389): 0 hour and 1 hour
  - p-4EBP1(S65): 1 hour and 3 hours
  - p-S6(S235/236): 1 hour and 3 hours
  - p27: 1, 3, 12, and 24 hours
  - p-Rb(S780): 3 hours and 24 hours
- Lanes 1–6 for p-Foxo3a(T32) in the T47D (*PIK3CA* H1047R) panel in Fig 2A appear similar to lanes 1–6 for p-Foxo1(T24) in the BT474 (HER2-amp, *PIK3CA* K111N) panel in Fig 3A.
- In Fig S1B, after adjusting color levels, there appears to be horizontal discontinuity left and right from the band for p-Akt(T308) in lane 3.

The corresponding author’s response did not resolve the background discontinuity concerns observed in Fig 1A. Regarding the similarities observed between Figs 2A and 3A, the corresponding author stated that the published Fig 2A T47D panel is incorrect and provided a replacement panel from the original experiment. Editorial assessment of the replacement panel raised additional concerns. The corresponding author stated that original blots for the other panels of concern could not be retrieved. They provided repeat experiment data, but PLOS does not consider that repeat experiment data are sufficient to resolve concerns pertaining to the integrity of the published results.

In light of the above concerns that question the reliability and integrity of the results presented in [1], the *PLOS One* Editors retract this article.

QBS, SC, QY, HEH, and NR did not agree with the retraction and stand by the article’s findings. JL, KMH, KRL, and DDFJ either did not respond directly or could not be reached.
